# Feasibility of Imaging-Based 3-Dimensional Models to Design Patient-Specific Osteosynthesis Plates and Drilling Guides

**DOI:** 10.1001/jamanetworkopen.2020.37519

**Published:** 2021-02-18

**Authors:** Frank F. A. IJpma, Anne M. L. Meesters, Bram B. J. Merema, Kaj ten Duis, Jean-Paul P. M. de Vries, Hester Banierink, Klaus W. Wendt, Joep Kraeima, Max J. H. Witjes

**Affiliations:** 1 Department of Surgery, University of Groningen, University Medical Center Groningen, Groningen, the Netherlands; 2 3D Lab, University of Groningen, University Medical Center Groningen, Groningen, the Netherlands; 3 Department of Oral and Maxillofacial Surgery, University of Groningen, University Medical Center Groningen, Groningen, the Netherlands

## Abstract

**Importance:**

In acetabular fracture surgery, achieving an optimal reconstruction of the articular surface decreases the risk of osteoarthritis and the subsequent need for total hip arthroplasty. However, no one-size-fits-all osteosynthesis plate is available owing to differences in fracture patterns and variations in pelvic anatomy. Currently, osteosynthesis plates need to be manually contoured intraoperatively, often resulting in inadequate reduction and fixation of the fractured segments.

**Objective:**

To determine the feasibility and accuracy of a novel concept of fast-track 3-dimensional (3-D) virtual surgical planning and patient-specific osteosynthesis for complex acetabular fracture surgery.

**Design, Setting, and Participants:**

This case series study examines the use of patient-specific osteosynthesis plates for patients needing operative treatment for displaced associated-type acetabular fractures at a tertiary university-affiliated referral center and level 1 trauma center between January 1, 2017, and December 31, 2018. Models were created in 3-D based on computed tomography (CT) data, fractures were virtually reduced, and implant positions were discussed in a multidisciplinary team of clinicians and engineers. Patient-specific osteosynthesis plates with drilling guides were designed, produced, sterilized and clinically applied within 4 days. Data were analyzed at the 1-year follow-up.

**Exposures:**

Development and clinical implementation of personalized fracture surgery.

**Main Outcomes and Measures:**

The primary outcome was the quality of the reduction as determined by the postoperative CT scan. The secondary outcomes were accuracy of the screw placement and clinical outcome using patient-reported outcome measures.

**Results:**

Ten patients with a median (range) age of 63 (46-79) years with an acetabular fracture were included. The median (interquartile range [IQR]) preoperative gap was 20 (15-22) mm, and the median (IQR) step-off was 5 (3-11) mm. Postoperatively, the median (IQR) gap was reduced to 3 (2-5) mm (*P* = .005), and the median (IQR) step-off was reduced to 0 (0-2) mm (*P* = .01), indicating good fracture reduction, indicating good fracture reduction. The mean difference between the preoperative and postoperative gap was 14.6 (95% CI, 10-19) mm, and the mean difference in step-off was 5.7 (95% CI, 2-9) mm. The median (IQR) difference in screw direction between the planning and actual surgery was only 7.1° (7°-8°). All patients retained their native hip and reported good physical functioning at follow-up.

**Conclusions and Relevance:**

These findings suggest that 3-D virtual surgical planning, manufacturing, and clinical application of patient-specific osteosynthesis plates and drilling guides was feasible and yielded good clinical outcomes. Fast-track personalized surgical treatment could open a new era for the treatment of complex injuries.

## Key Points

**Question:**

What is the feasibility of using a personalized approach for reconstruction of complex fractures?

**Findings:**

In this case series study, 10 patients with severe acetabular fractures were surgically treated using 3-dimensional (3-D) surgical planning, patient-specific osteosynthesis plates, and drilling guides. These were designed, produced, sterilized and clinically applied within 4 days and were associated with an accurate reconstruction of the fractured articular surface and a good 1-year clinical outcome.

**Meaning:**

These findings suggest that 3-D virtual surgical planning, manufacturing, and clinical application of patient-specific osteosynthesis plates and drilling guides were feasible and yielded good clinical outcomes.

## Teaser

This case series study examines the use of a 3-dimensional virtual surgical planning and patient-specific osteosynthesis for complex acetabular fracture surgery.

## Introduction

The overall incidence of acetabular fractures is estimated as 5 to 8 per 100 000 people per year, which accounts for approximately 60 000 injured individuals annually in Europe.^1,2^ A pelvic injury can have major consequences for physical functioning, participating in social activities, and the ability to work. Acetabular fracture treatment consists of either nonoperative treatment (56% of patients), open reduction and internal fixation (38% of patients) or primary total hip arthroplasty (THA; 6% of patients).^3^

Achieving an optimal reconstruction of the articular surface is associated with improved functional outcome and decreased risk of progressive osteoarthritis and subsequent need for THA,^4,5,6^ but this is particularly challenging for associated-type (ie, more complex) fractures, according to the Letournel-Judet classification, with substantial displacement.^5,7,8^ Postoperative computer tomography (CT) analysis of acetabular fractures has demonstrated inadequate reductions in up to 53% of patients in a study by Verbeek et al.^5^ Additionally, 36% of patients with an inadequate reduction in the study by Verbeek et al^5^ eventually need a conversion to THA, compared with only 10% of patients with an adequate reduction.

Despite the progress in surgical techniques and osteosynthesis plates, even experienced surgeons often do not achieve adequate reconstruction of the fractured acetabulum.^5^ Currently, conventional osteosynthesis plates often require multiple intraoperative contouring maneuvers to fit the individual pelvis reasonably. Moreover, the optimal screw positions with good purchase might be hard to determine and verify with fluoroscopy. Unfortunately, a uniform osteosynthesis plate that fits the shape of each pelvis, covers all the fracture patterns, and holds the surgically reduced fracture fragments perfectly in place does not exist.

We developed an innovative surgical procedure for acetabular fractures by using 3-D virtual surgical planning and custom-made patient-specific pelvic osteosynthesis plates with drilling guides.^9^ We hypothesized that this new personalized approach would be associated with optimal osteosynthesis plate fitting, accurate screw placements, and adequate fracture reductions. The patient-specific osteosynthesis plates, tailored to both the shape of the pelvis and the fracture type, could be applied to repair accurately one of the most challenging fracture types in orthopedic trauma surgery. Therefore, the aim of this study was to assess whether such a personalized approach is feasible and was associated with accurate reconstruction of associated-type acetabular fractures.

## Methods

Approval for this case series was obtained from the University Medical Center Groningen institutional review board. All patients provided written informed consent. This study is reported following Strengthening the Reporting of Observational Studies in Epidemiology (STROBE) reporting guideline.

### Patients

Eligibility criteria were patients who sustained a displaced unilateral associated-type acetabular fracture^10,11^ and required surgical treatment. All consecutive patients with comminuted, displaced T-shaped, or both-column acetabular fracture types between January 1, 2017, and December 31, 2018, were included. A total of 10 patients were treated with patient-specific osteosynthesis plates according to the fast-track personalized procedures (eFigure 1 in the Supplement).

### 3-D Surgical Planning

**Figure 1.**
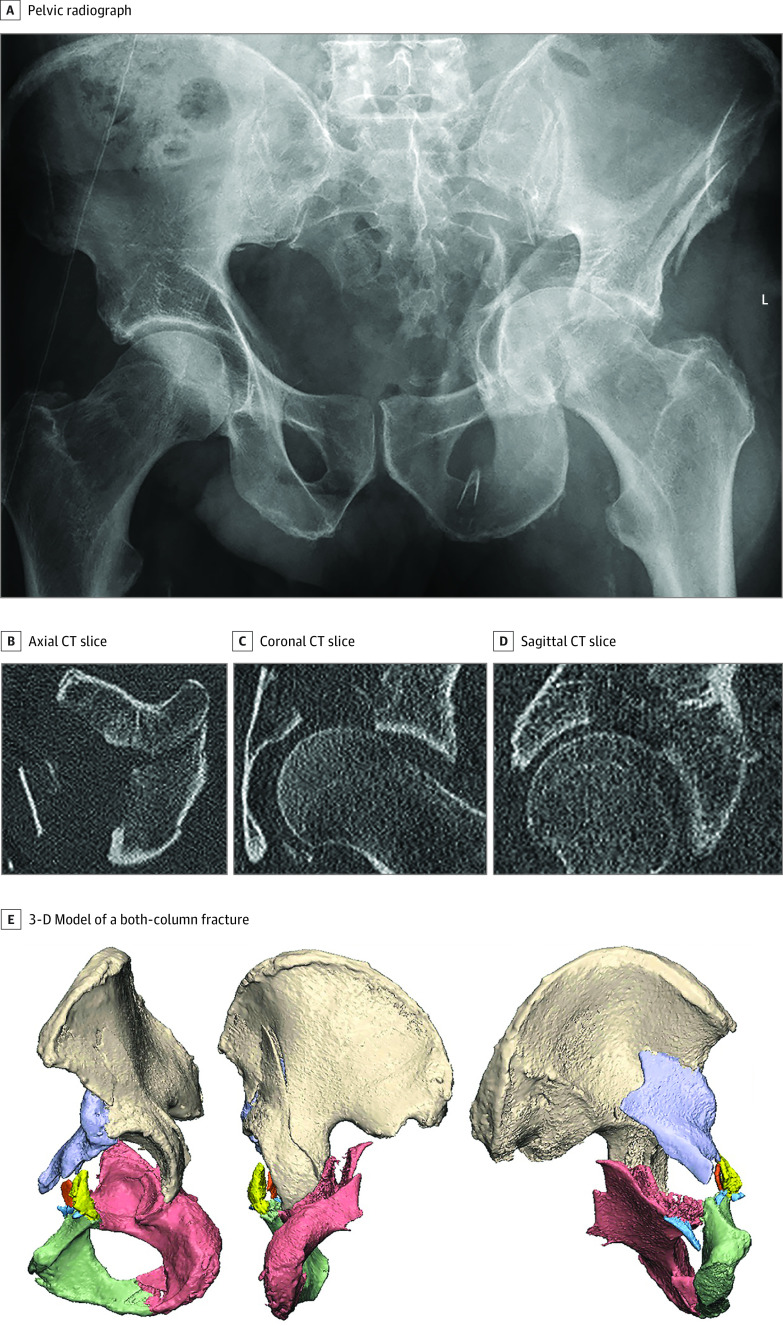
Preoperative Situation Pelvic radiograph, CT-scan (axial, coronal, sagittal CT slices) and 3-D model from patient 5, who had fallen from a height and sustained an associated-type both-column fracture. The fracture caused medial protrusion of the femoral head and severe displacement of the anterior as well as the posterior column. This patient was treated with patient-specific osteosynthesis plates.

Each patient’s CT data (≤1 mm slices; spatial resolution, 0.5-0.6 mm) was used to create a 3-D model (Figure 1) using Mimics Medical software version 19.0 (Materialise). A preset threshold for bone was used for automatic segmentation of all the fracture fragments. Each fracture fragment was assigned a different color and reduced to its anatomical position by using translational and rotational tools in the planning software. The contralateral hemipelvis was mirrored and used as a template to verify the accuracy of the virtual fracture reduction.^12^

### Patient-Specific Osteosynthesis Plate Design

**Figure 2.**
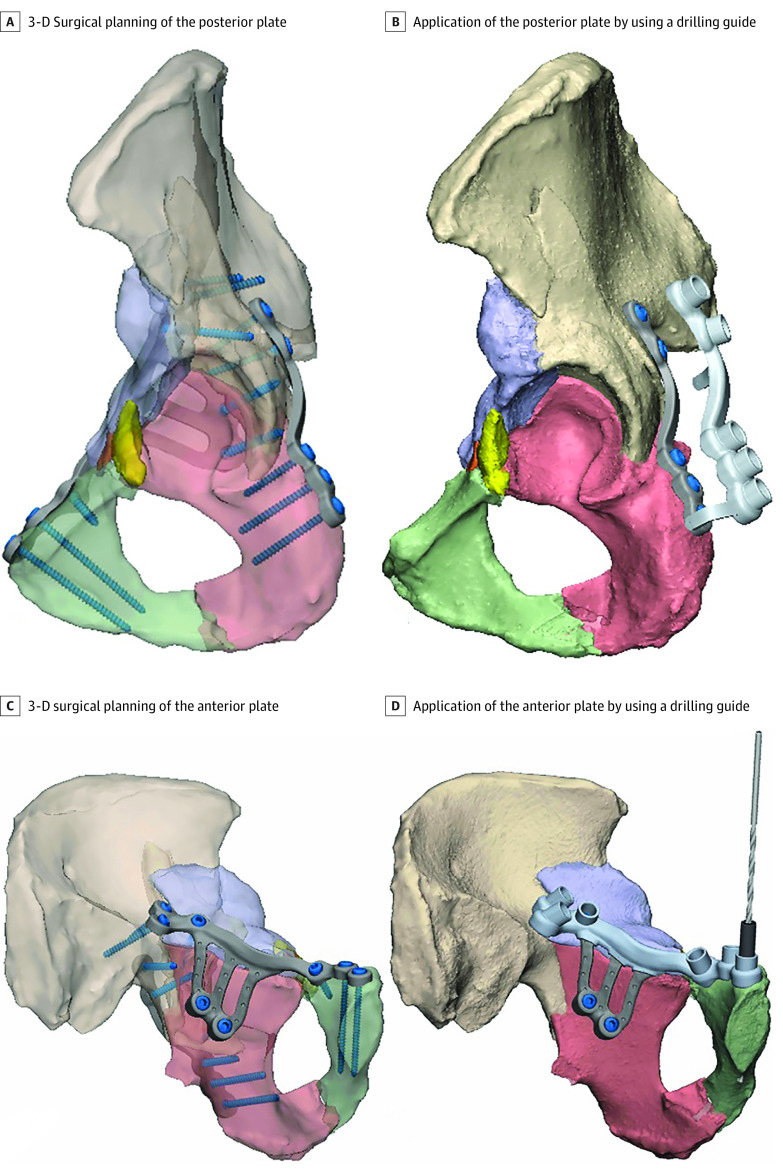
3-D Virtual Surgical Planning These images represent the preoperative planning, osteosynthesis plate designs, and drilling guides for patient 5, who underwent an associated-type both-column fracture operation. Our plan was to first perform a Kocher-Langenbeck approach, reduce the posterior column with a collinear reduction clamp and apply the patient-specific posterior plate (A). The proximal screws in the posterior plate were aimed at the ilium (cream-colored fragment) by using the drilling guide (B) to not compromise the reduction of the anterior column (purple fragment). After reduction and fixation of the posterior column, the wound was closed, and an additional anterior intrapelvic approach was performed. The anterior column was stabilized with a patient-specific anterior plate (C), which provided optimal support along the quadrilateral surface (red fragment). The screws in the anterior plate were aimed in the right direction by the drilling guide placed temporarily on top of the osteosynthesis plate (D).

Patient-specific osteosynthesis plates tailored from the virtually reduced 3-D fracture model, to achieve optimal support and stable internal fixation of fracture fragments, were designed within 1 day after hospital admission. First, the optimal screw trajectories in relation to the fracture fragments were predetermined in the 3-D fracture model. Subsequently, the patient-specific titanium osteosynthesis plates were designed with 3-Matic software version 11.0 (Materialise), Solidworks Professional software, version 2017 (Dassault Systèmes Solidworks), and the Geomagic package for Solidworks (3D Systems). All screw lengths were predetermined as part of preoperative planning. Drilling guides were designed to translate the virtual planning to the surgical procedure and to guide the drill bit as well as the screws in the right direction (Figure 2). The guides were provided with additional bone supporting extensions that enabled a perioperative visual check for correct placement. In addition, the guides were supplied with holes for k-wire fixation once the correct position was achieved. The synthetic guides were designed to envelope the osteosynthesis plate and to allow insertion of a stainless steel drill sleeve while drilling the screw pilot holes. The surgical plan, screw trajectories, and osteosynthesis plate design were discussed in a multidisciplinary meeting with pelvic surgeons (F.F.A.IJ. and K.t.D.), technical physicians (A.M.L.M. and J.K.), and an engineer (B.M.).

### Osteosynthesis Plate Production Process

The custom-designed patient-specific osteosynthesis plates were manufactured by a regional medical company (Witec Medical) within 3 days for each patient. The osteosynthesis plate was milled out of a medical grade titanium alloy by a 5-axis milling machine. The drilling guides were 3-D laser-printed from medical certified polyamide powder (Oceanz). The osteosynthesis plates and guides were prepared for surgery with a routine 134 °C autoclave steam sterilization process.

### Surgical Procedure

**Figure 3.**
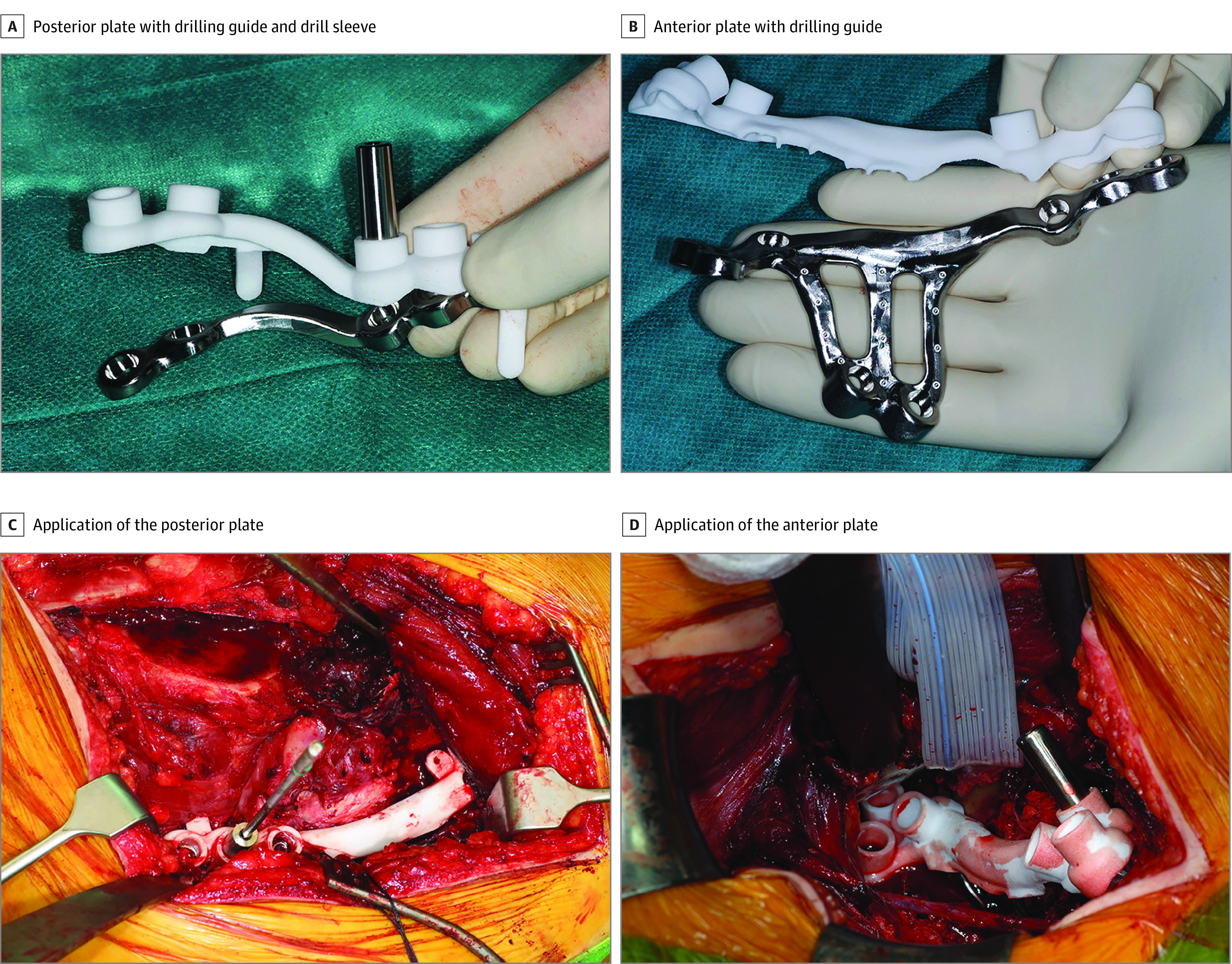
Intraoperative Situation Intraoperative images of the patient-specific posterior and anterior osteosynthesis plates used to treat the acetabular fracture of patient 5. A, posterior plate, including the drilling guide and drill sleeve. B, anterior plate, including the drilling guide. C, intraoperative reduction of the posterior column through a Kocher-Langenbeck approach by using a collinear clamp and the subsequent application of the posterior plate with the corresponding drilling guide. D, application of the anterior plate by using an anterior intrapelvic approach.

All the patients underwent operations according to the standard of care by 2 trauma surgeons (F.F.A.IJ. and K.t.D.), each with more than 5 years of experience in pelvic surgery, which helped to avoid bias of the results due to differences in operative skills between surgeons. The fracture pattern and amount of displacement were decisive for the surgical approach. The most suitable approach (ie, anterior intrapelvic approach with or without a lateral window, Kocher-Langenbeck, or a combined approach) was left to the treating surgeon and discussed before designing the osteosynthesis plate. After exposing the fractures, standard tools were used to perform fracture reduction, during which patient-specific osteosynthesis plates were applied to put or keep the fracture fragments in place. The drilling guides with additional bone-supporting extensions, which enabled visual checks when positioning the osteosynthesis plate, were placed on top of the osteosynthesis plate (Figure 3). Intraoperative fluoroscopy was used to verify that the osteosynthesis plate was positioned according to the preoperative 3-D planning. A drill sleeve was inserted into the cylinders of the guide to aim the drill bit correctly. After drilling, the drill sleeve was removed and screws of a predetermined length were inserted 1 by 1 into their proper trajectory. After inserting the last screw, the fracture reduction and osteosynthesis material were checked with fluoroscopy. The drilling guide was removed from the osteosynthesis plate, and all wounds were closed in layers.

### Postoperative CT Evaluation

**Figure 4.**
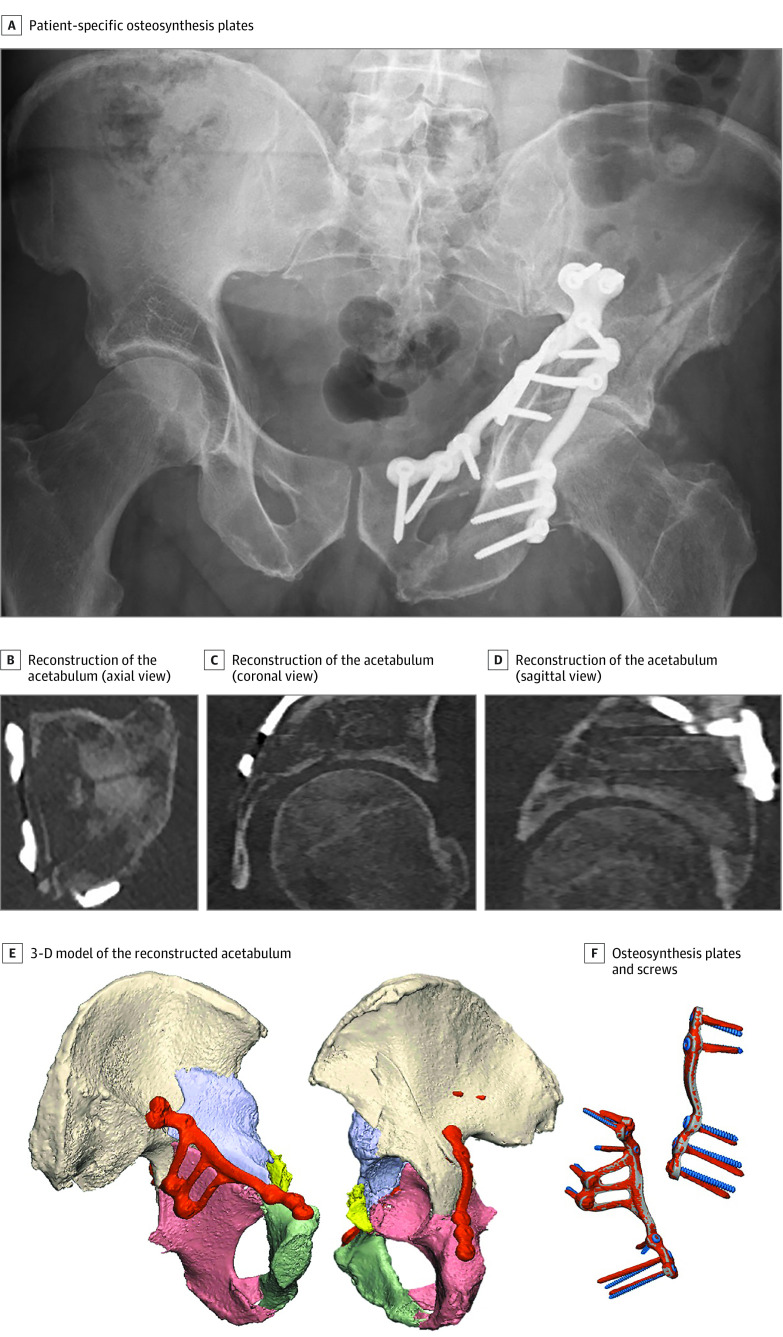
Postoperative Situation Patient-specific osteosynthesis plates were placed in patient 5 according to the preoperative plan. A postoperative computed tomography (CT) scan demonstrated good reconstruction of the fractured acetabulum. The gap was reduced from 28 mm before the operation to 5 mm after the operation. The step-off was reduced from 15 mm before the operation to 2 mm after the operation. A 3-dimensional (3-D) model, generated from the postoperative CT data, demonstrated an accurate surgical reconstruction of the fractured acetabulum (bottom left and middle). The osteosynthesis plates and screws, retrieved from the postoperative CT scan (orange), were digitally matched with the preoperative planning (gray and blue, F). The matched osteosynthesis plates demonstrated that all screws had been inserted accurately with only a median deviation of 6.8 degrees between the preoperative planning and the actual execution. The patient recovered uneventfully and returned to work after 6 months of rehabilitation. At the 1-year follow-up, the patient had no complaints about the hip, with no pain or physical impairment.

A postoperative CT scan (≤1 mm slice thickness) was performed to evaluate the accuracy of the acetabular reconstruction and screw positioning (Figure 4). Two experienced trauma surgeons (F.F.A.IJ. and K.t.D.) who were blinded to patient data assessed the quality of the reduction by measuring the greatest residual gap or step-off displacement at the acetabular dome on the postoperative CT scan in any of the axial, sagittal, or coronal plane views according to a standardized method.^13^ The quality of the reduction was graded according to Matta criteria and the newly proposed CT-based criteria.^8,14,15^

Furthermore, postoperative CT data were used to create a 3-D model of the reconstructed pelvis, which was matched using surface-based matching with the preoperative 3-D surgical planning to assess how accurately the preoperative plan had been executed (Figure 4). The deviation in screw direction between the preoperative and postoperative 3-D model was determined for each screw. Additionally, differences in screw lengths between the preoperative planning and postoperative situation were calculated to assess the feasibility of using preplanned screw lengths and of thereby saving some operation time.

### Clinical Follow-up

All patients were followed up at 3 months and 1 year. The patient-reported outcomes were assessed at the time of admission (preinjury score) and during both visits with the validated Short Musculoskeletal Function Assessment (SMFA) questionnaire. The SMFA evaluates the functional status of a patient with various musculoskeletal disorders and injuries. It consists of 46 items regarding physical function of the extremities, daily activities, and mental or emotional problems. The SMFA scores may vary from 0 to 100, with a higher score indicating a worse function.^16,17^ Also, date of return to work was recorded. All complications were monitored during the follow-up.

### Statistical Analysis

The Wilcoxon signed rank test was used to assess differences between the preoperative and postoperative gap and step-off by using SPSS statistical software version 23 (IBM). Furthermore, the paired samples Wilcoxon signed rank test was used to assess differences between the preinjury, 3-month, and 1-year patient-reported outcome scores (SMFA scores). The Spearman correlation coefficient was used to assess correlations between the postoperative reduction (ie, gaps and step-offs) and clinical outcome (SMFA). *P* values were 2-sided, and statistical significance was set at *P* = .05. Data were analyzed at the 1-year follow-up.

## Results

### Demographic Characteristics

Ten patients, including 9 men and 1 woman, with a median (range) age of 63 (46-79) years who sustained an associated-type both-column fracture or T-shaped fracture were included. A total of 15 patient-specific osteosynthesis plates were used. Five patients were treated with only an anterior plate, and the other 5 patients received an additional posterior plate (Table).

**Table.** Patient Characteristics

Patient No.	Age, y	Sex	Trauma mechanism	Fracture type	Surgical approach	Osteosynthesis plates
1	40s	Man	Fell off bicycle	Both-column	AIP + LW + KL + TF	Ant + post
2	60s	Man	Fell off bicycle	T-shaped	AIP + KL	Ant + post
3	60s	Man	Fall from height	Both-column	AIP + LW	Ant
4	70s	Man	Fell off scooter	Both-column	AIP + LW	Ant
5	60s	Man	Fall from height	Both-column	AIP + KL	Ant + post
6	40s	Man	Fell off bicycle	Both-column	AIP	Ant
7	40s	Man	Fell off bicycle	Both-column	AIP	Ant
8	60s	Man	Fall from height	Both-column	AIP	Ant
9	70s	Man	Fall from height	T-shaped	AIP + KL	Ant + post
10	70s	Woman	Fell off bicycle	Both-column	AIP + KL	Ant + post

Abbreviations: AIP, anterior intrapelvic; ant, anterior; LW, lateral window; KL, Kocher-Langenbeck; post, posterior; TF, trochanter flip.

### Surgical Procedure

The osteosynthesis plates and drilling guides were designed, fabricated, and sterilized within 4 days (Video). All plates fit well and did not need additional bending maneuvers during the operation. All the patient-specific osteosynthesis plates were a good reference for the reduction and functioned as guides for fracture reduction. Only 3 of 95 screws that were preoperatively planned could not be placed during surgery owing to the narrow space and the presence of soft tissues deep in the pelvis, which hampered the predetermined drilling angle. None of the screws penetrated into the hip joint or caused soft tissue injuries.

**Video.** Acetabular Fracture Repair Using Patient-Specific Osteosynthesis Plates and Drilling Guides Illustration of 3-dimensionalsurgical planning; patient-specific osteosynthesis plate and drilling guide development; surgical procedure; and postoperative computed tomography and 3-dimensional modeling evaluation.

### Postoperative CT Evaluation

Initially, the median (IQR) preoperative gap was 20 (15-22) mm, and the median (IQR) step-off was 5 (3-11) mm (eTable 1 in the Supplement). After patient-specific osteosynthesis plate surgery, the median (IQR) gap on the postoperative CT scan was reduced to 3 (2-5) mm (*P* = .005), and the median (IQR) step-off was reduced to 0 (0-2) mm (*P* = .01). The mean difference between the preoperative and postoperative gap was 14.6 (95% CI, 10-19) mm, and the mean difference in step-off was 5.7 (95% CI, 2-9) mm. According to CT-based criteria, the reduction was graded as perfect in 3 of 10 patients and good in 7 of 10 patients.^14^ The preoperative and postoperative axial CT slices at the acetabular dome are presented in eFigure 2 in the Supplement.

A total of 95 screws were placed by using a drilling guide. Per patient, the median (IQR) number of screws was 6.5 (6-7) anterior screws and 6 (5-7) posterior screws. The median (IQR) difference between the planned screw length and the actual screw length was 1.7 (1-3) mm, and the median (IQR) difference between the planned and actual screw direction was 7.1° (7°-8°), which are within the safe zone for using personalized acetabular fracture surgery with confidence in clinical practice.

### Clinical Follow-up

All patients retained their native hip at the 1-year follow-up. The median (IQR) preinjury SMFA function score was 7 (2-9), indicating good physical functioning (eTable 2 in the Supplement). Three months after surgery, the median (IQR) SMFA function score was 29 (22-35) and improved to 9 (5-27) at the 1-year follow-up (*P* = .04), indicating good physical functioning. The median (IQR) preinjury SMFA lower extremities score was initially 1 (0-8), then 30 (19-38) 3 months after surgery, and improved to 6 (3-19) at the 1-year follow-up (*P* = .04). There was no significant correlation between the SMFA scores at 1 year and the postoperative gap and step-off. At the 1-year follow-up, 3 patients reported almost the same level of physical functioning as before their injury, according to the SMFA questionnaire (eTable 2 in the Supplement). Four patients reported some decrease in physical function at the 1-year follow-up despite an accurate operative reconstruction of the fractured acetabulum. These patients were older (aged 62 to 79 years), with some comorbidity and preexisting functional impairment. Of these 4 patients, 2 patients reported better physical functioning at 3 months than at 1 year, owing to a progression in secondary arthrosis as seen on the radiographs. Five patients returned to the same level of work as before their injury. Two patients partially returned to work, and 3 patients were already retired. One patient had a complication and needed readmission owing to a deep wound infection requiring multiple washouts and antibiotic treatment, after which he recovered, and the fracture healed. In 1 patient, the osteosynthesis plate was removed after 1 year at the patient’s request.

## Discussion

This case series demonstrates that the application of 3-D surgical planning and patient-specific osteosynthesis plates, combined with drilling guides, was feasible and allowed for accurate operative reconstruction of complex acetabular fractures within 4 days after trauma. The application of patient-specific osteosynthesis plates and drilling guides provides the possibility to execute the preoperative plan and attain the predetermined osteosynthesis plate and screw positions, which were associated with accurate reconstruction of the articular surface and good functional patient recovery.

The use of 3-D virtual models allows the surgeon to gain more insight into the fracture pattern and treatment strategy.^18^ Moreover, 3-D-printed pelvic models are used as templates for fitting and precontouring conventional osteosynthesis plates before the actual surgical procedure.^18,19,20,21^ Several case series indicate that the use of 3-D-printed models in pelvic surgery are associated with reduced blood loss and shorter operation time.^22,23,24,25,26^ Although the reported use of prebent plates, adapted to printed 3-D–models, show some benefits, little data are available on whether 3-D–printed models improve the quality of the reduction.^22,23^ Our additional efforts in producing patient-specific osteosynthesis plates, with drilling guides, allowed for good alignment of the plate and screws to fit the fracture and shape variations of the pelvis, and using the plates as a reference was associated with good fracture reductions. A study comparing 3-D–printed model contouring and patient-specific osteosynthesis plates could reveal the indications for use per patient. Few preliminary reports about the clinical application of patient-specific osteosynthesis plates for pelvic fracture surgery are available to date. A study by Wang et al^27^ described the manufacturing of customized pelvic plates by using selective laser melting technology for 3-D metal printing and applied them to only 3 clinical cases. They did not investigate the accuracy of the fracture reduction or follow up the patients. A study by Xu et al^28^ used custom-made locking plates milled from titanium on 24 consecutive patients with acetabular fractures but did not use drilling guides as in our series. They reported some advantages, including the avoidance of intraoperative plate contouring, low risk of intraarticular screw penetration, low rate of osteosynthesis plate failure, and early mobilization of the patient. The main drawbacks of their series were the relatively long time required to produce the osteosynthesis plates (mean [SD], 6.9 [2.2] days) and the required technical demands. The study by Xu et al^28^ lacked a description of the performed placement accuracy, but the potential benefits are in line with our experiences. We managed to speed up the whole process, which provides opportunities for applying personalized fracture care on a larger scale in orthopedic trauma surgery. However, it is not possible to compare the current surgery results with those of Xu et al^28^ in terms of fracture reduction quality because of the differences in fracture types (severely displaced both-column fractures in our series vs all types of fractures in their series) and differences in imaging modalities used for postoperative assessment (CT scan vs radiograph).

Our techniques have some benefits for surgery. First, a review of the 3-D surgical planning by a multidisciplinary team provides an opportunity for consultation and the possibility to discuss the optimal surgical approach, features of the osteosynthesis plate, and screw positions, thus following the principle of “Plan your operation—and operate your plan!” as described by Schelkun.^29^ Second, guided screw placement enables tailoring the screw positions to the fracture reduction strategy. For instance, 1 patient sustained a severely displaced both-column fracture and needed a combined approach with posterior and anterior plates. The screws in the posterior plate were guided away from the anterior column and hence did not interfere with the reduction of the anterior column in the second phase of the operation. Also, the anterior plate was tailored to the fracture line in the ilium, which avoided an extra lateral window approach. Regarding another patient, the drilling guide was used to aim a lag screw through the anterior plate to indirectly reduce and fixate a large posterior wall fragment; screw accuracy planning was crucial here. Overall, the patient-specific osteosynthesis plates with guided screw insertion optimized fixation abilities and avoided additional surgical approaches in several patients.

### Future Perspectives

Over the next few years, we will work on increasing the efficiency of personalized fracture care. If software applications and advanced technologies contribute to more efficient and precise surgical treatment to the benefit of the patient by improving physical functioning for years, it would be worthwhile to explore the general applicability further in the near future. A follow-up study is needed that compares patient-specific osteosynthesis with conventional osteosynthesis.

### Limitations

This study has some limitations. One such limitation is that the advanced technologies are not applicable yet in all hospitals. We realize that these innovative techniques require sufficient resources, including the availability of dedicated engineers, validated software packages, and collaborative osteosynthesis plate production facilities. The cost for designing and producing the osteosynthesis plates was not part of this feasibility study. There is no selection bias, because in the interest of challenging our innovative personalized approach, the most complex acetabular fractures were eligible for this study. However, a potential confounding factor could be the experience of the surgeon. Therefore, all of the surgical procedures in this case series were performed by the same team. Another potential bias could be that the CT-based gap and step-off measurements are prone to intraobserver and interobserver variability.^30^ Therefore, measurements were performed in consensus by 2 experienced surgeons, who were blinded to patient data.

## Conclusions

In this case series, we describe the development and implementation of a patient-specific multidisciplinary workflow for acetabular fracture surgery that made it possible to reconstruct the acetabulum accurately and fixate the fracture fragments with custom-made osteosynthesis plates, resulting in good 1-year clinical outcome. Moreover, none of the osteosynthesis plates required intraoperative contouring maneuvers and all the screws could be placed accurately using the drilling guides.

## Notes

Supplement. **eTable 1.** Preoperative and Postoperative Displacement **eTable 2.** Clinical Outcomes Assessed by the Short Musculoskeletal Function Assessment Questionnaire **eFigure 1.** Timeline of the Workflow **eFigure 2.** Preoperative and Postoperative Computed Tomography Scans
