# Supplementary material for: Feasibility of Imaging-Based 3-Dimensional Models to Design Patient-Specific Osteosynthesis Plates and Drilling Guides
Source: JAMA Netw Open. 2021 Feb 18;4(2):e2037519. doi: 10.1001/jamanetworkopen.2020.37519 (PMC7893502; doi:10.1001/jamanetworkopen.2020.37519)
Supplement: Supplement. — eTable 1. Preoperative and Postoperative Displacement eTable 2. Clinical Outcomes Assessed by the Short Musculoskeletal Function Assessment Questionnaire eFigure 1. Timeline of the Workflow eFigure 2. Preoperative and Postoperative Computed Tomography Scans [file jamanetwopen-e2037519-s001.pdf]

Supplemental Online Content

IJpma FFA, Meesters AML, Merema BBJ, et al. Feasibility of imaging-based 3-dimensional models to design patient-specific osteosynthesis plates and drilling guides. *JAMA Netw Open*. 2021;4(2):e2037519. doi:10.1001/jamanetworkopen.2020.37519

eTable 1. Preoperative and Postoperative Displacement

eTable 2. Clinical Outcomes Assessed by the Short Musculoskeletal Function Assessment Questionnaire

eFigure 1. Timeline of the Workflow

eFigure 2. Preoperative and Postoperative Computed Tomography Scans

This supplemental material has been provided by the authors to give readers additional information about their work.

© 2021 IJpma FFA et al. *JAMA Network Open.*

eTable 1. Preoperative and Postoperative Displacement

Patient	Measurement	Preoperative	Postoperative
1	Gap<br>Step-off	21 mm<br>0 mm	2 mm<br>0 mm
2	Gap<br>Step-off	22 mm<br>12 mm	3 mm<br>0 mm
3	Gap<br>Step-off	19 mm<br>4 mm	5 mm<br>2 mm
4	Gap<br>Step-off	21 mm<br>3 mm	3 mm<br>0 mm
5	Gap<br>Step-off	28 mm<br>15 mm	5 mm<br>2 mm
6	Gap<br>Step-off	7 mm<br>0 mm	2 mm<br>0 mm
7	Gap<br>Step-off	9 mm<br>5 mm	3 mm<br>0 mm
8	Gap<br>Step-off	14 mm<br>4 mm	5 mm<br>2 mm
9	Gap<br>Step-off	23 mm<br>12 mm	4 mm<br>0 mm
10	Gap<br>Step-off	16 mm<br>8 mm	2 mm<br>0 mm
**Overall**<br>(Median, IQR)	Gap*<br>Step-off*	20 [15-22] mm<br>5 [3-11] mm	3 [2-5] mm<br>0 [0-2] mm

*: Significant improvement of the postoperative gap (P = 0.005) and step-off (P = 0.01).

© 2021 IJpma FFA et al. *JAMA Network Open.*

eTable 2. Clinical Outcomes Assessed by the Short Musculoskeletal Function Assessment Questionnaire

Patient	SMFA	Pre-injury	Three months follow-up	One year follow-up
1	Function Index	0.0	3.7	0.0
	Bother Index	0.0	0.0	0.0
	Lower Extremities	0.0	4.2	0.0
2	Function Index	7.4	20.6	10.3
	Bother Index	0.0	31.3	16.7
	Lower Extremities	2.1	18.8	6.3
3	Function Index	0.0	5.1	2.9
	Bother Index	0.0	2.1	2.1
	Lower Extremities	0.0	4.2	2.1
4	Function Index	18.4	43.4	30.1
	Bother Index	18.8	45.8	35.4
	Lower Extremities	12.5	47.9	20.8
5	Function Index	6.6	30.1	3.7
	Bother Index	4.2	37.5	0.0
	Lower Extremities	0.0	29.2	0.0
6	Function Index	7.4	33.1	8.1
	Bother Index	4.2	37.5	12.5
	Lower Extremities	6.3	33.3	6.3
7	Function Index	0.0	36.0	7.4
	Bother Index	0.0	33.3	10.4
	Lower Extremities	0.0	37.5	12.5
8	Function Index	15.4	35.3	40.4
	Bother Index	12.5	35.4	45.8
	Lower Extremities	14.6	37.5	43.8
9	Function Index	8.8	28.7	36.0
	Bother Index	10.4	27.1	50.0
	Lower Extremities	8.3	31.3	43.8
10	Function Index	8.1	27.2	19.1
	Bother Index	41.7	31.1	29.2
	Lower Extremities	0.0	20.8	6.3
**Overall** (Median, IQR)	Function Index	7.4 (2-9)	29.4 (22-35) *	9.2 (5-27) **
	Bother Index	4.2 (0-12)	32.3 (28-38)	14.6 (4-34)
	Lower Extremities	1.0 (0-8)	30.3 (19-38) *	6.3 (3-19) **

The SMFA scores may vary from 0 to 100, with a higher score indicating a poorer function.

**: Significant improvement in scores at one year compared to the scores at three months (P=0.04).*

***: Significant difference between pre-injury and one year scores (P=0.02).*

© 2021 IJpma FFA et al. *JAMA Network Open.*

eFigure 1. Timeline of the Workflow

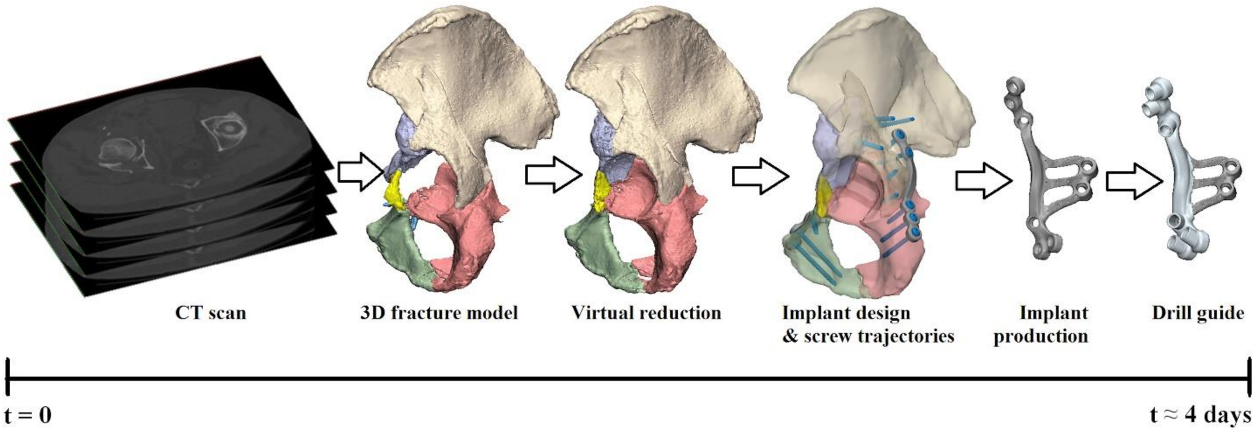

Workflow regarding the design and production of a patient-specific pelvic osteosynthesis plate tailored to the fracture type. A 3D model is generated from the CT data and the displaced fracture fragments are virtually reduced. The contralateral intact hemipelvis is used as a mirrored template in order to verify the accuracy of the reduction. The reduced 3D fracture model can be used to design and produce a patient-specific plate (milled titanium). A drilling guide (3D printed), designed to fit on top of the plate, is used to aim the screws in the right direction during surgery.

© 2021 IJpma FFA et al. *JAMA Network Open.*

eFigure 2. Preoperative and Postoperative Computed Tomography Scans

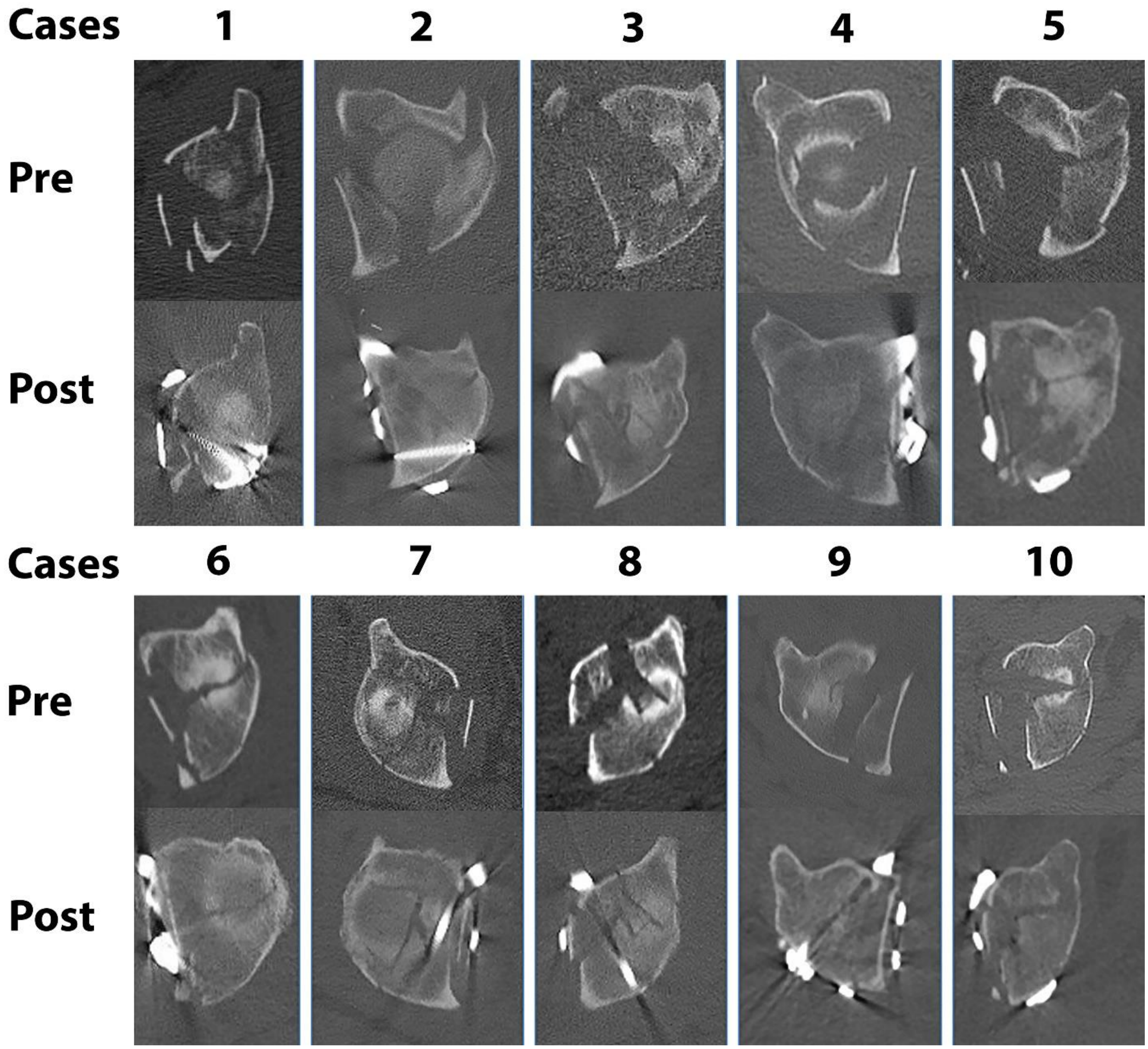

A comparison of the pre- and postoperative axial CT slices at the acetabular dome of all ten patients treated with patient-specific osteosynthesis plates. All the patients had multiple fracture lines and substantial displacement in multiple directions at the acetabular dome on the preoperative CT scan. After surgery, all the fractures were accurately reduced and fixated with patient-specific osteosynthesis plates.

© 2021 IJpma FFA et al. *JAMA Network Open.*
